# How much the Iranian government spent on disasters in the last 100 years? A critical policy analysis

**DOI:** 10.1186/s12962-020-00242-8

**Published:** 2020-10-19

**Authors:** Hamed Seddighi, Sadegh Seddighi

**Affiliations:** 1 Student Research Committee, University of Social Welfare and Rehabilitation Sciences, Tehran, Iran; 2 Department of Mechanical Engineering, K. N. Toosi University of Technology, Tehran, Iran

**Keywords:** Policy analysis, Budgeting, Disasters, Resources allocation, Climate change

## Abstract

**Background:**

During the past 20 years, Iran has been experiencing a significant increase in the occurrence of disasters mainly due to the emergence of anthropogenic climate change. This paper aims at analyzing the trend of national budget allocation in Iran over the last 100 years to evaluate the focus of the Iranian state on the four phases of Preparedness, Mitigation, Response, and Recovery and propose modifications.

**Methods:**

It is used a critical policy analysis with what’s the problem represented approach. In this approach is focused on problematization and policy gaps. The most important policy statement in any government is the budget. During the first screening, 1028 regulations and laws were found from 1910 to 2020. After full text screening, 494 regulations and laws related to budget allocation to disasters were analyzed.

**Results:**

The Iranian government has spent around 29 billion USD on disasters during the last 100 years. Droughts, earthquake and flood have costs the government more than other disasters, accounting for more than 14, 6.9, and 6.1 billion USD, respectively, in the allocated budget. Most of the Iranian government expenditure during the last 100 years on various disasters such as drought, flood, earthquake, and COVID-19 has been spent on involuntary costs including Response and Recovery. Mitigation and Preparedness are the two critical disaster management phases with very small shares of national budgeting.

**Conclusions:**

From policy audit and policy gaps it is concluded that Iranian governments during last 100 years, problematized the issue of “disasters strike” and not “disasters’ risks”. In time of disasters, governments tried to solve the issues or impacts of disasters with budgeting to response and recovery. Nevertheless, disasters’ prevention or mitigation or preparedness was not a problem for Iranian governments from 1920 to 2020.

## Background

Anthropogenic climate change has led to more frequent natural disasters [1]. The frequency of disasters had an exponential growth through the last 100 years [2] affecting more people by disasters. Disasters cause can be classified into geophysical, hydrological, climatological, meteorological, biological, and technological disasters [3]. According to the Centre for Research on the Epidemiology of Disasters (CRED) [4] a disaster is “situation or event which overwhelms local capacity, necessitating a request at the national or international level for external assistance; an unforeseen and often sudden event that causes great damage, destruction and human suffering”. Climate-related disasters such as floods, storms, droughts, and heatwaves are responsible for 91% of disasters between 1998 and 2017 [5]. While the economic cost of disasters during the 1998 and 2017 period were 2908 billion USD globally, the share of climate-related disasters was 2245 billion USD, which accounts for 77% of the total disaster economic costs. Such costs are not the whole picture for disasters economic costs due to the lack of data for many disasters and the corresponding damages. Around 53% of the reported disasters are in high-income countries, while only 13% of the reported disasters are related to low income countries [4]. Climate-related disasters rose by 151% in the last two decades [4]. USA National Oceanic and Atmospheric Administration tracks disasters’ costs in this country [6]. In a study by NOAA’s National Centers for Environmental Information (NCEI) was found that 258 weather and climate disasters exposed more than $1 billion damage costs to USA [6]. The cumulative cost for these events exceeds $1.75 trillion. Billion-dollar events to affect the USA from 1980 to 2019 are drought, flood, freeze, severe storm, tropical cyclone, wildfire, and winter storm respectively [7]. The average annual cost of disasters in Australia between 1967 and 1999 was $1.75 billion [8]. The most costly disasters in this country during this time were flood, sever storm, and cyclone respectively [8].

Efficient disaster risk management (DRM) systems are vital in facing disasters requiring economic resources and human resources. DRM consists of four phases including mitigation, preparedness, response, and recovery [9]. In the mitigation phase, preparative activities are planned to minimize the impacts of disasters. Preparedness programs planned for enhancing the knowledge and capacity of the society at all levels are necessary to effectively respond to the disaster occurrence, and recover from the dire impact of disasters [10, 11]. For saving lives and reducing the health impacts of disasters, emergency services and public assistance should be organized. In the recovery phase, livelihood, facilities and living conditions of disaster-affected communities should be restored and be improved [12].

There were indicated numerous classification of costs associated with disasters in studies. According to the Sendai Framework, there are six indicators about disaster costs and losses including GDP losses due to disasters, agricultural losses, destroyed productive assets, housing sector losses, destroyed critical infrastructure, and damaged cultural heritage [13]. A survey by OECD (Organisation for Economic Co-operation and Development) for assessing the real cost of disasters showed the direct economic loss of critical infrastructure is the most significant indicator [14]. Stephenson et al. [15] categorized disasters costs to five type of losses including direct economic losses such as disruption of infrastructure costs; indirect economic costs (i.e. loss of jobs and production); direct non-monetary losses (i.e. loss of lives), indirect nonmonetary losses (i.e. disruption in social welfare); and loss of non-renewable natural resources (i.e. agricultural land). Parker et al. [16] divided the cost of disasters into three costs including direct cost, primary indirect costs, and secondary indirect costs. Altay et al. [17] classified disaster related costs into two categories including voluntary costs and involuntary costs. Expenditures on mitigation and preparedness costs are voluntary costs of disasters. Disaster response and recovery costs are considered involuntary type. Ideally, governments should allocate resources for disasters preparedness and mitigation [17, 18]. Also they should prepare themselves to allocate money in the time of disasters for response and post-disasters for recovery. These decisions for investment are the needed cost management framework [17]. A survey by OECD showed less than half of the survived governments, collected data and financially prepared for disaster risk management expenditures [14]. Those countries that collected data usually focused on a specific spending and not the whole expenditure. For instance, Australia mostly collected data on rehabilitation after disasters and France mostly focused on prevention expenditures. In addition, some countries use disaster-related data economic costs for other purposes such as disaster preparedness. For example, Japan used data on disaster damage cost to measure the effectiveness of their risk reduction programs before disasters, and the Australian government uses the data for risk communication [14]. There are challenges in identifying government expenditure on disasters. In various cases, governments do not reflect the disaster preparedness budgets in annual budgets or reports or in some cases such costs are embedded in other budget sections [14]. Identification of government expenditure on disasters provides a big picture of resources allocation to disaster management. Expenditures of the private sector on disasters will complete the picture required to analyze the disaster preparedness. Disaster costs are classified into the costs of four disaster phases including Disaster prevention and mitigation (i.e., hazard mapping, land-use planning, housing, resilience, risk awareness), Disaster preparedness (i.e. early warning systems, evacuation planning, emergency supplies, disaster education), disaster response (emergency supplies, relief items, cash transfer to affected people, search and rescue operation), and disaster recovery (i.e. recovery of public infrastructure) [14].

A good case study for disaster preparedness and resources allocation is Iran, which is afflicted by frequent natural disasters including floods, earthquakes, droughts, and sandstorms. After each disaster in the disaster-prone country of Iran, the government allocate budget to afflicted provinces. But, no report or study has been published on analyzing the governmental expenditures and governmental preparedness for disasters. The analysis of Iranian government policies for disasters can help policymakers to judge the consequences of resource allocation and planning on disaster preparedness. Budget analysis, and disaster phases’ costs reported in this work help the policymakers to analyze the effectiveness of cost analysis theory and cost allocation plans. The aim of this study is to investigate how Iranian governments in the last 100 years addressed disasters in the policies.

## To achieve the overall research purpose, six research questions guided document collection and analysis

1. How Iranian governments in the last 100 years allocated budget to disasters (Characteristics of budget allocation)?
2. What is the share of each disasters management’s phases (mitigation, preparedness, response, and recovery) in the budgeting?
3. Budgets were allocated to which disasters more?
4. How budgeting changed during the last 100 years in terms of disasters’ types and phases?
5. Where are the gaps in budget policies of Iranian governments in the last 100 years?
6. What might be the consequences of budgeting by Iranian governments in the last 100 years?

### Context

**Fig. 1:**
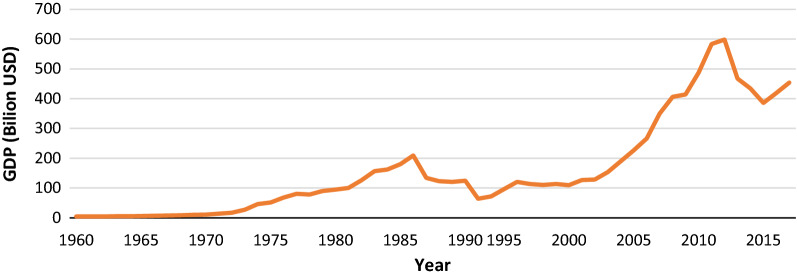
GDP per capita growth (annual  %)—Iran, Islamic Rep. from 1961 to 2017 (Data Source: World Bank)

Iran is a relatively large country in the Middle East region with a total area of 1,648,195 km and a population of 83,183,741 [19]. The World Bank estimated Gross Domestic Product (GDP) in 2017 of 447.7 billion USD [20]. Figure 1 shows the trend of GDP variations from 1960 to 2015. Iran has 31 provinces that governed by an appointed governors. The urban population is increased from 27% in 1950 to 74% in 2020 [19].

Iran is a disaster-prone country [21]. According to the world risk report [22], Iran is among the countries with high vulnerability in the world, which has resulted in significant impacts of disasters on the country including aspects such as financial, social, and physical losses. More than 80,000 people died during the last 30 years in Iran directly by natural disasters [23]. Iran’s provinces are located in the arid and semi-arid regions of the world. During Iran’s history, many natural disasters happened there, including extreme floods, extreme temperatures, and drought [24]. The country is also highly seismically prone, experiencing many tragic earthquakes [25]. Melville [26] studied frequent disasters in Iran in the last 1000 years, finding climate change has had a main role in happening frequent disasters including drought, earthquake, flood, Storm, heavy rainfall, hails storms, and sever winters [26]. While the above disasters are still threatening Iranian people, studies showed some regions of Iran are about to experience higher temperatures in the future [27]. Such temperature rises are leading to more frequent droughts which are a significant danger to the livelihood of rural families in Iran [28]. Another study regarding the pattern for flood magnitude and drought severity in Iran during 1950–2010 period, showed that the severity and magnitude of these two disasters are increasing [24]. Changes in land use, negative trend of annual rainfall, poor water resources management policies are the main causes of flood and drought growth [24]. Drought resulted in water-related disasters such as drying of lakes and rivers, dust storms, and dust haze [29].

## Methods

It is investigated in this study that whether and how Iranian governments have addressed disasters in budgeting involved a policy analysis. The most important policy statement in any government is the budget. There are rarely activities of government that do not need funds, and public funds should have spent with legislative authority. Budgets decide which services and policies are to be extended, reduced, lapsed, introduced, or renewed. The budget is at the core of all public policies. The budget process includes a framework to review government services, determine their expenses, compare them to financial capital, and make decisions between expenditures.

Answering to questions 1–4 those were mentioned in the introduction needed a budget policies audit via document collection. The questions 5–6 will be answered with a critical policy analysis.

### Document collection (policy audit)

Firstly, all Budget regulations related to disasters extracted from the “policies, laws, and regulations portal of the Islamic Republic of Iran” which is an organized portal under the legal deputy of the Iranian president. For retrieving the laws and statistics, the following terms were searched: floods OR hurricanes OR rain OR tornadoes OR volcanoes OR earthquakes OR tsunamis OR storms OR emergencies OR crisis OR hazards OR risks OR fire OR bushfire OR landslide OR haze OR sandstorm OR drought OR snow OR heatwave OR cold wave OR severe weather OR avalanche OR thunderstorms OR Red Crescent Society (current equivalent of red cross society) OR Red Lion and Sun Society (older equivalent of red cross society). During the first screening, 1028 regulations and laws were found from 1910 to 2020. After full text screening, 494 regulations and laws related to budget allocation to disasters were included and the remaining texts were excluded. The excluded regulations were not contained budget and only were about policies.

Five themes extracted from the laws and regulations including type of disasters, disaster phases, date, allocated budget, and Province. The language of legal documents is Farsi and the mentioned date is in the Persian calendar which was converted to the Gregorian calendar for the study. The allocated budget in the laws and regulations was in Iran’s currency Rial which was converted to US dollars for the sake of the study. The exchange rate of Rial to USD is taken from the Central Bank of the Islamic Republic of Iran and journals between 1924 and 2020. For comparing the costs of disasters in a range near to 100 years, the inflation of US dollars was considered using official records published by the U.S. Department of Labor. All budgets amounts in US dollars adjusted for inflation using the Consumer Price Index (CPI) for finding the annual inflation rate.

### Critical policy analysis with WPR approach

What’s the problem represented (WPR) is an approach to policy analysis that is introduced by Bacchi [30]. In the WPR approach, six questions about the problem are considered, including the problem itself, (2) presupposition for representation of the problem, (3) the problem’s origin, (4) policy silence, (5) effects produced by the representation of the problem, and (6) unproblematic issues. In this study, it is focused on three specific Bacchi’s questions including questions 1, 4, and 5. The first question of this approach is divided into this RQ’s 1–4, the fourth question is presented in RQ 5, and the fifth Bacchi’s question is shown in RQ 6.

In the traditional view, policies are reactions problems that should be solved. Conversely, governments do not respond in the WPR approach to problems which are believed to be self-evident. Rather, they are said to be interested in generating or developing “problems” as different kinds of problems, with specific criteria, causes, consequences, and solutions. This is believed that the policy proposals or suggested “solutions” by their definition contain tacit interpretations of the “problems” or “problems” that they claim to tackle. The WPR approach is a significant form of analysis in the whole field of practice research [31]. The concentration in this approach is on establishing which interventions work to address which problems. This approach provides useful insights into the modes of governing. Rose and Miller (1992, p. 181) indicated that “government” is a “problematizing activity” [32]. Osborne (1997, p. 174) discussed that “policy cannot get to work without first problematizing its territory” [33]. That is, in order for something to be governed, or imagined as governable, it needs to be problematized [34].

## Results

### Policy audit

The Iranian government spent around 29 billion USD ($29,637,146,774 USD) on disasters during the last 100 years. Eight disasters were mentioned in the allocated budget by the government, including drought, earthquake, flood, hurricane, sandstorm, severe weather, snow, and wildfire. Drought has costs for the government more than other disasters, accounting for more than 14 billion USD in the allocated budget. Earthquake and flood are two other major disasters in Iran in terms of government expenditure in 100 years with 6,970,711,164 USD and 6,142,587,944 USD, respectively. Table 1 shows the budget allocation to each disaster type in Iran during the last 100 years.

**Table 1:** Disaster budget allocation characteristics in Iran during the last 100 years

Disaster type	Frequency (Percent)	Cost (Percent)
All hazard	32 (6.49%)	$2,131,296,060 (7.19%)
Drought	95 (19.27%)	$14,149,803,467 (47.74%)
Earthquake	125 (25.35%)	$6,970,711,164 (23.52%)
Flood	232 (47.06%)	$6,142,587,944 (20.73%)
Hurricane	1 (0.20%)	$1,889,333 (0.01%)
Sandstorm	1 (0.20%)	$9,903,739 (0.03%)
Severe weather	3 (0.61%)	$44,632,952 (0.15%)
Snow	1 (0.20%)	$180,463,576 (0.61%)
Wildfire	3 (0.61%)	$5,858,539 (0.02%)
Total	493 (100%)	$29,637,146,775 (100.00%)

The allocated budget classified into four phases including Mitigation, Preparedness, Response, and Recovery.

The Response phase was the most expensive phase for the government and consumed almost half of the allocated budget to disasters during the last 100 years. Around 93% of the Response phase budget is allocated to drought and other disasters had only 7% share of the budget showing the shortcomings of the Iranian government in relation to drought Mitigation, Preparedness and Recovery phases.

The second phase in terms of disasters’ costs (40% of the budget) is the recovery. Around 98% of the Recovery phase budget is allocated to earthquake (48%) and flood (50%). The Preparedness phase has been the third priority for spending budget that 78% of the budget was allocated to all-hazard preparedness.

**Fig. 2:**
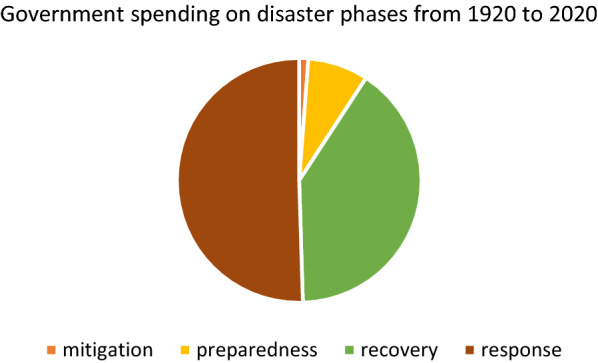
Government spending on disaster phases from 1920 to 2020

All Hazard approach shows while hazards are different in source (natural, human-made), their challenges for the society (health, economic, social) are same. Mitigation phase is the last phase and only 1% of the budget in the last 100 years was allocated to the Preparedness phase in Iran. Table 2 shows the government expenditure on disasters regarding the type of disasters and disaster phases. Figure 2 shows that most of the budgets are allocated to response and recovery phases.

**Table 2:** Disaster phases, budget allocation from 1920-2020

Type	Total costs	Mitigation	Preparedness	Response	Recovery
All-hazards	$2,131,296,060	$66,378,878	$1,847,736,510	$173,365,462	$43,815,211
Drought	$14,149,803,467	$0	$0	$14,045,892,103	$103,911,363
Earthquake	$6,970,711,164	$20,704,176	$473,041,250	$715,967,340	$5,760,998,397
Flood	$6,142,587,944	$90,207,062	$0	$12,040,824	$6,040,340,058
Hurricane	$1,889,333	$0	$0	$0	$1,889,333
Sandstorm	$9,903,739	$0	$0	$9,903,739	$0
Severe weather	$44,632,952	$0	$44,487,195	$0	$145,757
Snow	$180,463,576	$180,463,576	$0	$0	$0
Wildfire	$5,858,539	$0	$1,320,499	$2,887,417	$1,650,623
Total	$29,637,146,774	$357,753,693	$2,366,585,454	$14,960,056,886	$11,952,750,742

**Fig. 3:**
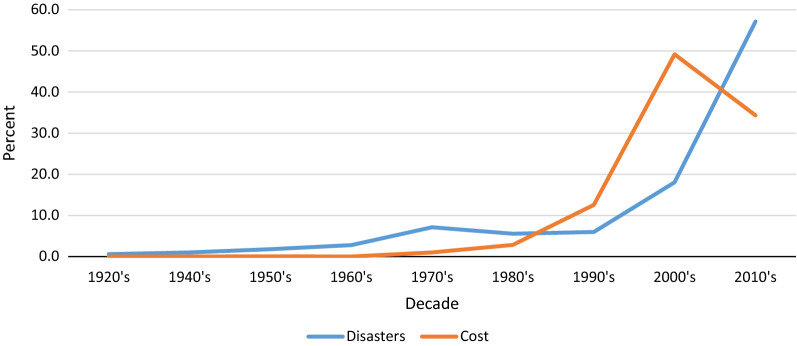
Frequency and cost of disasters (percentage)

Figure 3 shows that with increasing in disasters, The Iranian government spent more on disasters from 1920’s to 2000’s. However, from 2010, despite of the increasing frequency of disasters, fewer budgets were allocated to disasters. The reduced budget allocation relative to increasing disasters frequency from 2010 can be motivated by the crippling effects of US sanctions on the Iran economy starting from 2009.

**Fig. 4:**
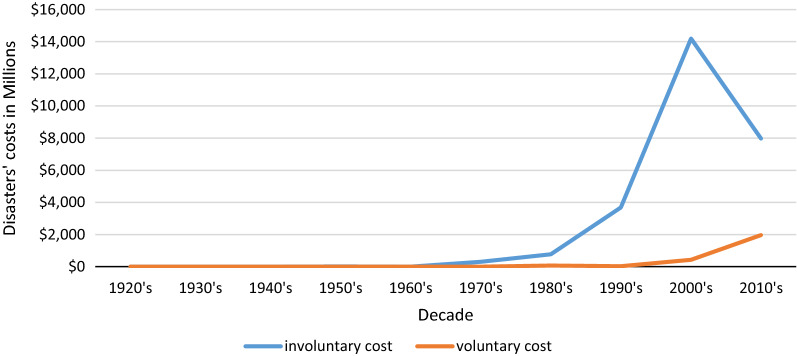
Voluntary and involuntary cost of disasters in Iran from 1920 to 2020

Figure 4 shows that involuntary cost of disasters (response and recovery) for the Iranian government is extremely higher than the voluntary cost of disasters (mitigation and preparedness). Voluntary costs of disasters were mostly allocated to preparedness budgets for emergency organizations such as Iranian Red Crescent Society and Iranian Crisis Management organization. Mitigation budgets were allocated to some ministries for drought, earthquake, and flood mitigation and were distributed nationally. Involuntary costs of disasters mostly distributed between affected provinces.

**Fig. 5:**
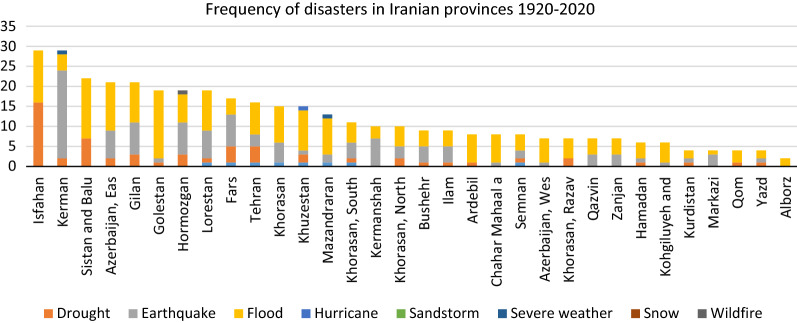
Frequency of disasters in Iranian provinces 1920–2020

While based on allocated budgets during 100 years, the major budget allocation has gone into drought, earthquake, and flood, the pattern of disasters in each province is naturally different. Figure 5 shows the frequency of disasters in each province. Isfahan has the highest frequency of disasters mainly due to the drought and flood. Kerman has the second highest frequency of disasters in Iran mainly due to the earthquakes.

**Fig. 6:**
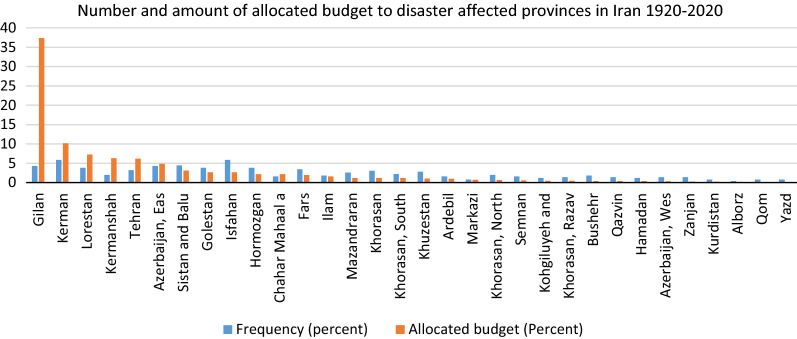
Number and amount of allocated budget to disaster affected provinces in Iran 1920–2020

Figure 5 shows frequency of disasters in different provinces of Iran during the last 100 years. While Isfahan and Kerman have had the highest frequency of disasters, Gilan province has had the highest disaster budget allocation followed by Kerman province (Fig. 6).

### Policy gaps

The finding of this study shows that mitigation and preparedness have had the least share of the government disasters expenditure in Iran in the last 100 years. The Sendai Framework for Disaster Risk Reduction 2015–2030 emphasized on four priorities for disasters prevention including “(i) Understanding disaster risk; (ii) Strengthening disaster risk governance to manage disaster risk; (iii) Investing in disaster reduction for resilience and; (iv) Enhancing disaster preparedness for effective response, and to “Build Back Better” in recovery, rehabilitation and reconstruction” [35]. However, the budget analysis performed in this work shows that the Iranian policies have not aligned with the priorities suggested by the Sendai Framework for Disaster Risk Reduction 2015–2030. Iranian government mostly spent on disasters response and recovery. This type of disaster budget allocation performed in Iran, is inefficient and results in a constantly decreasing share of mitigation and preparedness from the governmental resources [36]. Many researchers mentioned that the burden of costs related to disasters, particularly disaster recovery, must be covered by the governments [37]. Many disaster-related costs are necessary for restoring public services and assets. Restoring moral expectations and emotional situations are also among the costs which have not been predicted in budgets but are necessary to maintain a healthy society [14, 38]. Generally, increasing investment on pre-disaster activities such as Mitigation and Preparedness phases is more cost-effective than post-disaster programs [39, 40]. Many governments, including most of OECD countries, focused on the protection and resilience of critical infrastructures. In OECD countries, there are identified twenty-two policy tools for strengthening critical infrastructures [41].

From government expenditure on earthquakes, 82% allocated to the Recovery phase and 10% allocated to the Response phase. Earthquakes are sudden-onset disasters and are not predictable. Hence, many studies conducted on earthquakes in Iran and furthermore, earthquake prone regions in Iran are found by seismologists [42–47]. With various studies on earthquakes, it is expected to see more investment and expenditure on earthquake mitigation. This expectation has not been met in the Iranian budget, even in the last decades with development in technology, science, and GDP. More investment on prevention and preparedness will improve response and recovery. According to the Iranian Parliament report (2019), the government failed in response and recovery of the last major earthquake in Kermanshah in 2017, even with a significant budget allocation [48]. In this study, using budget regulations analysis in Iran during the last 100 years, it is found that more than 98% of flood budget allocations have been for the flood Recovery phase. The significant point in flood risk management is mitigation. Policy gap in mitigation approaches to disasters including flood are clear.

## Discussion

The trend of natural disasters in Iran in the last 100 years follows the global natural disasters trend. Globally, while from 1900 to 1980, fewer than 100 disasters are reported annually, the number of disasters in the period of 2000 to 2019 is between 300 and 400 disasters annually [49]. Natural disasters that were reflected in Iran’s budget allocation regulations have the same trend where both disaster frequency and the associated budget significantly rose until 2009. The increase in the frequency of natural disasters can be motivated by improved reporting by governments in addition to the emergence of anthropogenic climate change. The findings of this study show that the most frequent natural disasters in Iran in the last 100 years are floods, earthquakes, and droughts. According to Centre for Research on the Epidemiology of Disasters (CRED) [49], between 1970 and 2019, floods are the most frequent disaster in the world, followed by extreme weather conditions, droughts, earthquakes, landslides, and wildfires.

Floods are the most frequent climate-related disasters in the last 100 years in Iran, according to budget regulations and the second in the amount of budget (20%). Floods usually bring mass destruction associated with economic, physical, and social losses. Neglecting floods for governments is very difficult mainly because of the social pressure. Nevertheless, it could be hypothesized that the statistics of floods in the budget regulations are true. In Iran, heavy temporary rainfall, and in some regions, the combination with sudden snow melt are the main reasons for flooding. Heavy rainfall especially happens in the North of Iran (north of Alborz Mountains), Southwest (Zagros Mountain), south (near Persian Gulf), and South East (The Hirmand river) [29]. From 2015 to 2020, eight major floods occurred in Iran [29, 50–52]. The worst flood in Iran in a recent decade was in 2019 that affected to 26 provinces of Iran out of the total 31 provinces [51]. A flood in 2001 in Golestan province in the north of Iran was another major flood [29]. Floods are more predictable compared to other disasters, and mitigation and prevention have significant roles in risks reduction. For example, there were many articles discussing flood prone regions in Iran before the great flood in 2019 [53–58] which could have used by the Iranian government to prevent such high damages. However, mass destruction of infrastructure and allocating a significant amount of budget to flood affected provinces in 2019, is a proof for underestimating flood risks by the government [59].

According to the findings, earthquakes are the second disasters in both of frequency (25%) and budget (23%) during the last 100 years. West of Iran (along the Zagros collision) is earthquake prone because of the collision of Arabian and Eurasian plates. In addition, the south of Iran is along the Makran subduction is also seismically active [60]. Examples of tragic earthquakes in Iran can be seen in Qazvin earthquake in 1962, Khorasan earthquake (1948), Khakhk earthquake in 1968, Dashti Biaz earthquake in Khorasan province (in 1931,1941,1947, and 1962), Tabas earthquake (1978), Manjil–Rudbar earthquake in 1990, Bam earthquake in 2003, Kermanshah earthquake in 2017 [61–64]. In the budgeting in Iran during the last 100 years, two earthquakes were more significant, including Manjil–Rudbar earthquake in 1990 and Bam earthquake in 2003. As shown in Fig. 4, there are two jumps in government expenditures in 1990’s and 2000’s. These two jumps are related to two earthquakes in Manjil (1990) and Bam (2003).

Droughts are the most expensive disasters for the Iranian government in the last 100 years and more than 47% of disasters costs belongs to droughts. Drought is referred to water resources shortage over a large geographical area in a significant period [65]. Iran is an arid and semi-arid country. Despite of deserts, other regions of Iran are experiencing an increase in climate change toward less precipitation [29]. Drought is the most complex disaster and the least understandable one [66]. Iran is struggling with droughts for the decades, and therefore Iran must take key steps in addressing droughts. Most of expenditures related to droughts are dedicated to response. Nevertheless, in comparison to other disasters such as flood and earthquake, its frequency and visible devastation are slow, leading to less sensitivity of mass media and people. In contrast to other disaster types, there are not reliable reports from losses resulted from drought (economic, socially, physically). Thus, the reliability of drought budget allocation is very difficult. Two provinces including Isfahan and Sistan & Baluchistan gained drought-related budgets compared to other provinces. Many provinces in Iran, such as Yazd and Far, have been suffering from droughts during the last decades in the similar scale as the Isfahan and Sistan provinces [66]. For example, Yazd province, which is one the most drought-affected regions in Iran, is in the bottom of the list in terms of drought budget [67]. Unfortunately, budget allocation for drought disasters, is mainly related to political issues such as members of Iranian parliament, the share of this province in the power hierarchy, security issues, and media. Thus, an integrated energy-water-food analysis and management plans (such as that performed in [68]) are necessary in water management plans and drought mitigation plans in Iran.

In terms of disaster frequency based on budget allocation, top five provinces are Kerman (5%), Isfahan (5%), Gilan (4%), East Azerbaijan (5%), and Sistan Baluchistan (5%). However, it is not true to conclude that these provinces necessarily have experienced more disasters than other provinces. For instance, Gilan province experienced several floods during the last 100 years in addition to a devastating earthquake in 1990 that the Iranian government allocated a large budget for the response and recovery of that earthquake [69]. Kerman is the second province in terms of disaster budget allocation mainly because of earthquake frequency in this province. An enormous earthquake occurred in Bam city in Kerman province in 2003 is one of the deadliest earthquakes in this province that killed more than 26,000 [70].

Due to climate change, the current resources such as facilities, water consumption policies and technologies, and construction standards are not enough to response to disasters under climate change such as drought, flood, and extreme weather. Thus, Iran must invest more into preparing it infrastructures for the coming disasters [71]. More importantly, the disasters stemmed from climate change are not solo events. Climate change related disasters are strongly coupled and act as dominos. For example, drought and heatwave occur together. Drought leads to dry soils and as a result, solar energy from evaporation will end to increase surface warming and consequent increased evaporation rates [72]. Drought and heatwave will increase the risk of wildfires. Furthermore, sandstorms, haze, and water conflicts are other consequences of drought. Iran, like many other countries, has a disaster management plan as an isolated event and a solo hazard, while it should be managed as cascading hazards [72]. A cause of increasing cascade effects of major disasters is critical infrastructure failures such as transportation, water supply, energy, and communication [14]. Infrastructure and hazards are correlated and failure in providing through infrastructures enhances disasters’ impacts while disaster lead to failure in infrastructures. This interrelationship makes a vicious cycle or trap that if not addressed properly, its impacts will be bigger and bigger like an avalanche in many unprepared countries including the disaster-prone unprepared Iran.

## Conclusions

From the policy audit and policy gaps, it is concluded that Iranian governments during the last 100 years, the problematized issue of “disasters strike” and not “disasters’ risks”. In the time of disasters, governments tried to solve the issues or impacts of disasters with budgeting to response and recovery. Nevertheless, disasters’ prevention or mitigation or preparedness was not a problem for Iranian governments from 1920 to 2020. This “problematization” for solving disasters’ issues was part of problems in next disasters because, it resulted in less expenditure on mitigation and consequently, more devastation by later disasters. It seems, Iranian government should change their problematization to disaster risks. It is necessary a comprehensive and all hazard approach for disaster risk reduction in the Iranian policies.

## Data Availability

The datasets used and/or analysed during the current study are available from the corresponding author on reasonable request.

## Abbreviations

CRED: Centre for Research on the Epidemiology of Disasters
USA: United States of America
NOAA: National Oceanic and Atmospheric Administration
NCEI: National Centers for Environmental Information
DRM: Disaster risk management
GDP: Gross domestic product
OECD: Organisation for Economic Co-operation and Development
RQ: Research question
CPI: Consumer Price Index
USD: US dollar
WPR: What’s the problem represented
